# Personal

**Published:** 1897-10

**Authors:** 


### Personal.

Dr. L. A. Weigel, of Rochester, has established an office at 199 Franklin street, Buffalo, where he can be consulted on Wednesdays only in relation to orthopedic surgery, to which practice he devotes his exclusive attention. Consultation hours, 10 to 1. Telephone, Seneca 287.

Dr. William G. Bissell, bacteriologist to the department of health and lately assistant surgeon 74th Regiment, N. G. N. Y., has been promoted to be surgeon of that regiment. It is wise for the National Guard to retain the services of such efficient officers as Dr. Bissell and to promote them whenever vacancies exist.

Dr. Roswell Park, of Buffalo, who is convalescent from a prolonged illness, sailed for the Mediterranean, September 25, 1897. He was accompanied by Mrs. Park, and expects to be absent about three months, when he will return and resume his professional and college work.

